# Organizational Culture at a Tertiary Care Center in Saudi Arabia: A Mixed Approach Study

**DOI:** 10.7759/cureus.3736

**Published:** 2018-12-15

**Authors:** Ghassan Abass, Ali Asery, Mohamad Al-Tannir, Humariya Heena, Isamme AlFayyad, Ahmed Al-Badr

**Affiliations:** 1 Miscellaneous, King Fahad Medical City, Riyadh, SAU; 2 Pediatrics, King Fahad Medical City, Riyadh, SAU; 3 Epidemiology and Public Health, King Fahad Medical City, Riyadh, SAU; 4 Obstetrics and Gynecology, King Fahad Medical City, Riyadh, SAU

**Keywords:** organizational culture, employees perspective, leadership behavior

## Abstract

Objective

To explore the prevailing culture among leaders at the King Fahad Medical City (KFMC) in Riyadh, Saudi Arabia, as well as to evaluate the impact of organizational culture from the perspective of employees.

Subjects and methods

Utilizing a convenient sampling method, data were collected at the KFMC, Saudi Arabia, using two research approaches (focus group and cross-sectional). A total of 446 employees participated in this questionnaire-based study, and all questionnaires were analyzed to obtain the final data.

Results

About 51% of the participants were males and 49% were non-Saudi nationals. On a scale of 5 points, the overall score of the KFMC culture assessment was 3.6 (72%). The strongest recognized factor was the enjoyment (3.9/5; 78%). On the other hand, the factor with the lowest score was innovation (3.3/5; 66%).

Conclusion

The culture within an organization is very important. Communicating organizational strategic plans of change to employees and assuring their acknowledgment of the vision can inspire the behavior and attitude of employees at the workplace. This study strikes a note of challenge in some dimensions and items. Top management leaders are recommended to adjust their leadership behavior to focus on these challenges.

## Introduction

Over the last few decades, several studies have been done in the arena of organizational culture (OC) across various industries, including healthcare organizations. OC can be defined as a set of shared and recognized values, beliefs, or perceptions among the employees or members of an organization [1]. A proper understanding of OC is important for implementing strategies that benefit an organization, as well as for effectively managing the increasing and changing demands of the marketplace [2]. In any big organization, two types of cultures may exist, primary dominant culture and many subcultures. The dominant culture defines the perception or opinion shared by most of the employees of an organization. In contrast, subcultures mainly originate as a result of difficulties or situations experienced by the employees within the organization. According to Cameron and Quinn, four kinds of OCs are there: tribal, adhocracy, market-based, and hierarchical cultures. Tribal culture gives more importance to the flexibility over supervision and control, whereas market-based culture stresses more on the supervision and control than flexibility. Adhocracy culture concerns more on extra-organizational issues, innovations, freedom for decision-making, and flexibility. In contrast, hierarchical culture is associated more with intra-organizational issues [3]. In addition to improving the working efficiency and environment, a good OC also brings work satisfaction, which, in turn, is associated with higher overall organizational productivity [2]. A recent study has demonstrated that poor OC and job satisfaction are significantly related to a high rate of absenteeism and associated economic burdens [4]. Thus, OC definitely plays an important role in maintaining the teamwork's effectiveness and job satisfaction, which together affect the provider’s performance [5].

In order to bring productive changes to an organization, it is sometimes important to transform an OC into a learning organization to meet the requirements of the demand-supply chain [6]. Organizational learning is a critical factor, especially in healthcare settings, because a high-quality therapeutic outcome mainly depends on the provider's performance. In this regard, a dynamic learning culture is an important aspect of establishing a successful performance management program [7]. A number of organizational learning practices and aspects of learning culture, such as motivation to bring effective changes, encouraging open discussion, and creating learning-oriented goals rather than performance-oriented goals, are associated with improved patient care in health management systems [8-9]. Studies have also shown that organizational learning cultures are linked with lower rates of adverse events and readmission [10].

Apart from establishing a good learning culture, introducing and promoting innovation is one of the major aspects in a healthcare organization to meet the ever-growing demands and expectations of patients as well as to maintain a cost-effective medical facility [11]. However, setting up innovation in an organization is a complex process, which can be achieved through organizational strategies, including the development of incentives, improvement in knowledge, promoting inter-disciplinary collaborations, and establishing infrastructure for innovation [12].

Therefore, to efficiently reform a healthcare organization and suitably utilize its resources, establishing a good OC is essential. An accurate evaluation of key factors that may affect the work culture of an organization is, therefore, necessary to implement and manage its overall productivity.

King Fahad Medical City (KFMC) embarked on an umbrella project that aimed at achieving cultural transformation. To ensure the success of this project, a necessary pre-requisite was to identify the characteristics of the existing organizational culture by the organization's leaders. Therefore, the present survey aimed to explore the prevailing KFMC culture among leaders and to identify the magnitude of the impact of OC from the perspective of employees.

## Materials and methods

Study setting and participants

The study was conducted at the KFMC in Riyadh, Saudi Arabia, using both qualitative and quantitative approaches. All KFMC leaders were enrolled in the qualitative phase, and all KFMC employees were included in the quantitative phase.

Study Design

In the qualitative phase, a focus group technique was employed that involved KFMC's first-line leaders exploring and identifying the core dimensions and criteria items at the KFMC. In this context, the most prevailing 10 dimensions of the KFMC culture are as follows:

1.     Professionalism: Referred to as the normative utilization and reflection of communication, values, clinical intellectual, knowledge, and skills in everyday practice [13].

2.     Organizational Learning (Develop Continuously): Referred to as the ongoing process of creating, retaining, and transferring knowledge within the organization [14].

3.     Integrity (Openness and Honesty): Referred to as complying with an organization’s ethical commitments with openness and honesty on a daily basis.

4.     Respect: Referred to as treating others fairly and impartially without discrimination and harassment and with proper respect for their rights and obligations.

5.     Managing Change: Referred to as the extent to which the management and executives of the organization bring changes so that employees can cope with them.

6.     Goals Integration: Referred to as the extent to which the individual and departmental goals are in line with the overall organizational goals, as well as bring a proper understanding of such goals among employees.

7.     Patient (Customer) Orientation: Referred to as matching particular, internally derived, and defined goals with patients’ needs and views.

8.     Cultural Strength: Referred to as a group of values and beliefs strongly shared by most or all of the organization's members for the greater stability of its productivity.

9.     Enjoyment: Referred to as the outlook of employees regarding coming to the workplace and their perception of an enjoyable workplace.

10.  Innovation: Referred to as the capacity to harness intellectual and social capital to convert it into novel ideas.

In the quantitative phase, using a cross-sectional study design, a self-administered questionnaire (two-language version: Arabic and English) was designed to assess the extent of OC impact from the perspective of employees. Before the main fieldwork, a back-translation was performed, followed by verification b consultants to validate the quality of the translation. Due to cultural considerations, some non-substantive items and questions were modified and some were excluded (e.g., the question about ethnicity). Survey items were adapted from the itemsets obtained through a collaboration with a research corporation. Cronbach’s alpha for different item sets was reported to be above 0.7.

Data collection/instrument

The questionnaire comprised two sections. The first section consisted of participants’ demographic information, including age, gender, job group, and nationality. The second section included the participants’ perception of OC. The items included in each dimension are as follows:

First Dimension: Professionalism (six items): Employee’s contribution to the organization's goals, their good qualifications, an appreciation of their knowledge, admiring their endeavor for the excellence, pursuing personal objectives at the organization's expense, and the KFMC management strongly seeking professional expertise.

Second Dimension: Organizational Learning (six items): The importance of learning, work environment encouraging reasonable risk captivating, sharing knowledge and expertise, employee investments, and improving KFMC core operations and capabilities to be successful.

Third Dimension: Integrity (eight items): Awareness of ethical and integrity standards, reporting misconduct, considering standards of integrity and participating in decision-making, importance of integrity and performing right things, violation of integrity standards by employees, improper use of organizational resources, making false or misleading promises to others, and presenting false or misleading information to the public.

Fourth Dimension: Respect (six items): Mutual respect, absence or inappropriate social behavior, policies and guidelines for managing workplace harassment and discrimination, guidance on appropriate workplace behavior, mechanisms for dealing with workplace bullying, harassment, and discrimination, and expression of political, religious, or social views at the workplace.

Fifth Dimension: Managing Change (six items): Flexibility and adaptability for changes, feelings toward changes imposed by the top management, why and how to proceed throughout the process of change, applying gradual changes without causing excessive disruption, employee’s influence at the workplace through ideas and involvements, and concerns and anxieties during the period of change.

Sixth Dimension: Goal integration (six items): The goals defined by the employees that relate to the organization’s mission, reaching attainable goals, measured and rewarded works according to goal achievement, participating in defining specific goals, and stretching goals to improve organizational image continuously and consistently to the outside world.

Seventh Dimension: Patient Orientation (six items): Priority and support in meeting patients’ needs and solving their problems, policies, and procedures describing required services for patients, recognizing patients' problems, new ways to improve patient services, recognizing or rewarding employees for providing the best patient service, and making improvements using patients’ feedback.

Eighth Dimension: Cultural Strength (four items): Value and use of colleagues' strengths and abilities, compromising organization policies and procedures to reach operational goals, decision-making by facts and not just perceptions or assumptions, and timely access to accurate information about incidents in the organization.

Ninth Dimension: Enjoyment (four items): Employees work well when they enjoy work, employees enjoy the company of colleagues, employees stay late to finish a certain work that interests them, having fun interferes with getting work done, employees smile and greet each other, and employees enjoy among themselves.

Tenth Dimension: Innovation (seven items): New ideas are set forth, exploring alternative approaches to solve problems, supporting innovative employees, innovation opportunities and new ideas are given a try, failures are quickly forgotten, and minimum red tape for new ideas.

The survey utilized a forced-response approach, leading to the elimination of all missing data. The assessment of dimensions was done using the following scoring guideline: A score of 0-39 was considered poor, 40-59 was considered fair, 60-79 was good, and 80-100 was excellent.

Ethical consideration

Institutional Review Board approval was obtained from the KFMC-IRB office. The identity of each participant was maintained confidentially.

Statistical analysis

All data were analyzed using SPSS version 21.0 (SPSS, Chicago, IL, USA). Descriptive statistics (mean, standard deviation, and percentages) were used to describe the quantitative and categorical variables. A thematic analysis and categorization were used to identify the prevailing dimensions of OC.

## Results

A total of 446 participants completed the questionnaire. The demographic characteristics of the participants are summarized in Table 1. The percentages of the participants from allied health services, administration, nursing, physician, and operation departments were 43, 34, 10, 9, and 4, respectively.

**Table 1:** Demographic characteristics of the respondents

Characteristics	Number (n=446)	Percentages (n%)
Age		
18-25 year	27	6%
26-35 year	187	42%
36-45 year	161	36%
46-55 year	58	13%
56-65 year	13	3%
Gender		
Male	227	51%
Female	219	49%
Nationality		
Saudi	192	43%
Non-Saudi	254	57%
Profession group		
Physician	40	9%
Nursing	44	10%
Administrative professional	152	34%
Allied Health Services	43	43%
Operational service	4	4%

The descriptive analysis performed using the survey data for assessing different dimensions showed a mean score of 3.6 (72%), indicating a good perception level about the KFMC culture among study participants. Among the studied dimensions, enjoyment received the highest score of 3.9 (72%), whereas innovation received the lowest score of 3.3 (66%). Regarding other dimensions, professionalism, managing change, cultural strength, integrity, organizational goal integration, organizational learning, respect, and patient orientation received scores of 3.7 (74%), 3.4 (68%), 3.5 (70%), 3.5 (70%), 3.6 (72%), 3.7 (74%), 3.7 (74%), and 3.7 (74%), respectively. 

Regarding the professionalism dimension, the item “All employees are expected to make the best contribution possible towards the organization's goals” received the highest score of 4.5 (90%). The mean score for this dimension was 3.7 (78%), indicating a good level of professionalism in the KFMC culture.

Regarding the organizational learning dimension, the item “KFMC has the capability to be successful today and in the future” received the highest mean score of 4.1 (82%), whereas the item “Employees believe that they are being invested in” received the lowest score of 3.3 (66%). The overall mean score for this dimension was 3.7 (74%), demonstrating a good level of organizational learning culture at the KFMC.

Regarding the integrity dimension, two items, including “Employees do not improperly use organizational resources (e.g. intranet, office supplies or equipment)” and “Employees do not make false or misleading promises to others,” received the highest score of 3.8 (76%). In contrast, the item “Staff report misconduct when they see it” received the lowest score of 3.2 (64%). The overall mean score for this dimension was 3.5 (70%), indicating a good integrity level in the KFMC culture.

Regarding the respect dimension, the item “Employees do not express political, religious or social views at the workplace that offend other staff” received the highest score of 3.9 (78%). On the other hand, the lowest score of 3.5 (70%) was obtained against the item “The workplace is free of inappropriate social behavior (bullying, harassment, discrimination).” Overall, the mean score for this dimension was 3.7 (74%), showing a good level of respect among the employees at the KFMC.

Furthermore, in the case of the managing change dimension, the item “Employees are flexible and adaptable when changes are necessary” received the highest score of 3.8 (76%). In contrast, the lowest score of 3.2 (64%) was obtained against two items, including “Employees do not feel that change is the result of pressures imposed from higher up in the organization” and “Employees believe that their concerns and anxieties during periods of change are heard and taken into considerations.” However, the overall mean score for this dimension was 3.4 (68%), indicating a good perception level toward changes at the KFMC.

Regarding the organizational goal integration dimension, the item “Employees and teams are expected to reach goals which they believe are attainable” received the highest score of 3.9 (78%), whereas the item “Employees and teams works are measured and rewarded according to how well goals are achieved” received the lowest score of 3.1 (62%). The overall mean score for this dimension was 3.6 (72%), representing a good level of this dimension in the KFMC culture.

In case of the patient (customer) orientation dimension, the highest score of 4.0 (80%) was obtained against the item “The highest priority and support is given to meeting the needs of patients and solving their problems.” On the other hand, the lowest score of 3.1 (62%) was related to the item “Employees who do the best job of serving patients are likely to be recognized or rewarded.” The overall mean score for this dimension was 3.7 (74%), denoting a good level of patient (customer) orientation level in the KFMC culture.

In the case of the cultural strength dimension, the best-reported item was “employees value and make use of their colleagues' unique strengths and different abilities” with a mean score of 3.7 (74%); and the item with the lowest score of 3.2 (64%) was “Employees have access to timely and accurate information about what’s really happening in the organization and why.” The overall mean score for this dimension was 3.5 (70%), showing a good level of cultural strength at the KFMC.

Regarding the highest-rated dimension, enjoyment, the item that received the highest score of 4.4 (88%) was “Employees here work well when they are enjoying their work.” In contrast, the item “Employees enjoy themselves in this organization” with a score of 3.5 (70%) was indicated as the lowest-rated item. Nevertheless, the overall mean score for this dimension was 3.9 (78%), indicating a good workplace enjoyment level among the employees at the KFMC.

Regarding the lowest-rated dimension, innovation, the item “Employees thoroughly explore alternative approaches to problems” received the highest score of 3.8 (76%). On the other hand, the item “Employee’s failures are quickly forgotten” received the lowest score of 2.8 (56%). Overall, the mean score for this dimension was 3.3 (66%), indicating an average level of perception toward innovation among the employees at the KFMC.

## Discussion

The present study explored the leading dimensions of the KFMC culture as well as the perception of employees of the impact of these dimensions. This study provides significant new evidence on the changing cultural landscape for Saudi hospitals and links OC with overall performance in healthcare settings under the Saudi Ministry of Health.

Although several definitions are there to describe OC, it has been a difficult task to properly conceptualize OC because a wide range of dimensions can directly affect the working culture of an organization. Since the 1980s, several studies have been done to evaluate the culture of an organization. It is evidenced from these studies that two different approaches are there to define OC. According to the first approach, a company is known to have its own culture that helps employees become accustomed to the working environment of that particular organization; in contrast, the second approach defines OC as a system of knowledge that employees can interpret by their own. Such culture utilizes the benefits of knowledge enrichment through social interactions [15].

In the present study, a total of 446 participants from different departments at the KFMC were enrolled (Table 1). The overall score of the KFMC culture assessment was 3.6 out of 5 (72%), demonstrating a good level of perception among employees regarding the KFMC culture. The strongest recognized dimension was enjoyment (score 3.9/5; 78%). It is known that workplace enjoyment and the associated happiness increase the overall capacity of an organization to reach its goals [16]. Super-leadership has been shown to directly affect the workplace enjoyment among subordinates. A recent study involving German employees from different organizations has shown that two super-leadership aspects, including "coaching and communicative support" and "facilitation of personal autonomy and responsibility," have a direct influence on job enjoyment at the workplace [17]. A study describing the factors affecting the subjective well-being of social workers has stated that workplace happiness is directly influenced by intra- and inter-organizational relationships, the decision-making process, infrastructure and resources, workload, and the dynamics of management [18]. Organizational identification has also shown to regulate the effect of organizational justice on job satisfaction significantly [19]. The high level of inter-relationship between KFMC culture and staff satisfaction observed in our study strengthens the argument that supportive organizations give importance to their employees and promote their work enjoyment, which together increases the overall productivity.

On the other hand, innovation was identified as the weakest dimension (3.3/5; 66%) in the present study. Although the score reflects almost a good level of perception among employees, it may potentially impact the KFMC culture. However, items covered under this dimension and their respective scores are of great importance to identify the real problem and guide administrators to encourage both organizational learning and innovation because innovation is considered an important factor to bring productive changes within an organization [20].

Moreover, the item that received the highest score within the professionalism dimension was “All employees are expected to make the best contribution possible towards the organization's goals” (Table 2). This finding is a clear indication of an organization that is proactive toward deliberately planning strategies for achieving its shared goals. However, the lowest-rated items within this dimension call for attention toward appreciating and admiring specialized and knowledgeable employees for their excellence. In essence, these low-rated items may also affect the prospect of innovation and underpin the need for specific strategies to satisfy employee expectations [21].

The high score obtained for the learning dimension reflects a good overall perception of the employees toward learning and establishing necessary changes within the organization. It may also affect the innovation dimension positively at some point because organizational learning, along with global inter-disciplinary collaborative approaches, is a basic factor to initiate innovation within an organization [22].

Moreover, very good scores obtained for the integrity and respect dimensions reveal a high level of openness, ethical conduct, and overall honesty within the organization. Integrity is regarded as a combination of both personal and social morality, which can have serious impacts on an individual’s wellbeing and the trust culture of an organization [23-24]. Similarly, the respect among the employees and leaders is also an essential factor to establish a high-quality organization, which encourages employees to give their best toward the development of an organization [25].

Our study participants reported a flexible, adaptable, and positive experience in case of changes within the organization. A positive attitude toward changes is found to be important in attaining overall organizational goals and accomplishing changes [26]. Several studies have recommended that efforts to bring organizational changes can be a very traumatic and stressful experience for the employees [27]. However, participants in this study expressed that changes are not imposed on them from the top management; they also believed that their anxiety and stress resulting from the change are well considered and tailored by the administration. However, since the overall mean score for this dimension was found to be in the lower range, a strategic plan is recommended to maintain employees’ commitments toward changes within the organization. Moreover, participants showed good perception levels towards reaching attainable organization goals. This finding is interesting in terms of awareness of shared goals, the learning system to define specific goals, and participation to accomplish shared goals.

Regarding patient orientation, participants reported that meeting patients’ needs and solving their problem are highly prioritized and supported at the KFMC. They positively reported that patient’s feedback is utilized to make improvements within the organization. In this context, a previous study has revealed that non-managerial employees give more importance towards gathering and using knowledge to meet the need of a patient, whereas managerial employees prioritize learning orientation as a suitable way of improving the quality of care [28].

Regarding cultural strength, we infer that a good level of perception among the employees toward utilizing the unique strengths of colleagues reflects a collaborative and supportive working environment at the KFMC.

## Conclusions

The present study shows a good level of satisfaction among the employees at the KFMC. The organizational culture plays a significant role in creating a healthy and joyful working environment for its employees. Communicating organizational strategic plans of changes to employees and assuring their acknowledgment of the vision can inspire the behavior and attitude of the employees at the workplace. This study strikes a note of challenges in some dimensions and items. Top management leaders are recommended to adjust their leadership behavior to focus on these challenges.

## References

[1] Shahzad F, Luqman RA, Khan AR, Shabbir L (2012). Impact of organizational culture on organizational performance: an overview. IJCRB.

[2] Scammon DL, Tabler J, Brunisholz K (2014). Organizational culture associated with provider satisfaction. J Am Board Fam Med.

[3] Azizollah A, Abolghasem F, Dadgar MA (2015). The relationship between organizational culture and organizational commitment in Zahedan University of Medical Sciences. Glob J Health Sci.

[4] Llanos MR (2015). Job satisfaction and organizational culture as predictors of absenteeism [Article in Spanish, English]. Rev Med Chile.

[5] Korner M, Wirtz MA, Bengel J, Göritz AS (2015). Relationship of organizational culture, teamwork and job satisfaction in interprofessional teams. BMC Health Serv Res.

[6] Lindberg A, Meredith L (2012). Building a culture of learning through organizational development: the experiences of the Marin County Health and Human Services Department. J Evid Based Soc Work.

[7] Persaud D (2014). Enhancing learning, innovation, adaptation, and sustainability in health care organizations: The ELIAS Performance Management Framework. Health Care Manag.

[8] Mazanec P, Arfons L, Smith J (2015). Preparing trainees to deliver patient-centered care in an ambulatory cancer clinic. J Cancer Educ.

[9] Soklaridis SJ (2014). Improving hospital care: are learning organizations the answer?. Health Organ Manag.

[10] Wang X, Liu K, You L, Xiang J, Hu H, Zhang L, Zhu X (2014). The relationship between patient safety culture and adverse events: a questionnaire survey. Int J Nurs Stud.

[11] Maher L (2014). Building a culture for innovation: a leadership challenge. World Hosp Health Serv.

[12] Williams I (2011). Organizational readiness for innovation in health care: some lessons from the recent literature. Health Serv Manage Res.

[13] Epstein RM, Hundert EM (2002). Defining and assessing professional competence. JAMA.

[14] Argote L, Miron-Spektor E (2011). Organizational learning: from experience to knowledge. Organ Sci.

[15] Glisson C (2015). The role of organizational culture and climate in innovation and effectiveness. Hum Serv Organ Manag Leadersh Gov.

[16] Kerfoot KM (2015). The pursuit of happiness, science, and effective staffing: the leader’s challenge. Urol Nurs.

[17] Müller GF, Georgianna S, Schermelleh-Engel K (2013). Super-leadership and work enjoyment: direct and moderated influences. Psychol Rep.

[18] Shier ML, Graham JR (2013). Organizations and social worker well being: the intra-organizational context of practice and its impact on a practitioner’s subjective well-being. J Health Hum Serv Adm.

[19] Yuan G, Jia L, Zhao J (2016). Organizational identification moderates the impact of organizational justice on job satisfaction. Work.

[20] Goh S, Chan C, Kuziemsky C (2013). Teamwork, organizational learning, patient safety and job outcomes. Int J Health Care Qual Assu.

[21] Cleary M, Horsfall J (2013). Integrity and mental health nursing: factors to consider. Issues Ment Health Nurs.

[22] Crisp N (2014). Mutual learning and reverse innovation-where next?. Glob Health.

[23] Cleary M, Horsfall J (2013). Integrity and mental health nursing: factors to consider. Issues Ment Health Nurs.

[24] Pattison J, Kline T (2015). Facilitating a just and trusting culture. Int J Health Care Qual Assur.

[25] Leape LL, Shore MF, Dienstag JL, Mayer RJ, Edgman-Levitan S, Meyer GS, Healy GB (2012). Perspective: a culture of respect, part 2: creating a culture of respect. Acad Med.

[26] Eby LT, Adams DM, Russell JE A, Gaby SH (2000). Perceptions of organizational readiness for change: factors related to employees’ reactions to the implementation of team-based selling. Hum Relat.

[27] Elrod PD, Tippett DD (2002). The “death valley” of change. J Organ Change Manag.

[28] Bellou V (2010). The role of learning and customer orientation for delivering service quality to patients. J Health Organ Manag.

